# Left atrial appendage closure in a patient previously implanted with an interatrial shunt device: a case report

**DOI:** 10.1186/s12872-024-03904-0

**Published:** 2024-06-06

**Authors:** Dawei Lin, Mingfei Li, Zilong Weng, Wenzhi Pan, Daxin Zhou, Junbo Ge

**Affiliations:** 1 Department of Cardiology, Zhongshan Hospital, Fudan University, Shanghai, China; 2 National Clinical Research Center for Interventional Medicine, Shanghai, China; 3 Shanghai Institue of Cardiovascular Disease, Shanghai, China

**Keywords:** Left atrial appendage closure (LAAC), Interatrial shunt device (IASD), FreeFlow®, LAmbre device

## Abstract

Patients with previous interatrial shunt device (IASD) implantation may face greater challenges during future left atrial interventional procedures. Herein, we report the first case of left atrial appendage closure (LAAC) in a patient with previous IASD implantation. The patient successfully underwent LAAC using the LAmbre device without complications.

**Supplementary Information:**

The online version contains supplementary material available at 10.1186/s12872-024-03904-0.

## Introduction

In recent years, left atrial appendage closure (LAAC) has emerged as an effective operation strategy for patients with atrial fibrillation (AF) who are unsuitable for continuous oral anticoagulants [1–4]. Most of the formation of thrombosis occurs in left atrial appendages, especially in patients with AF [5–8]. Therefore, enclosing the left atrial appendage through a minimally invasive method with occluding device would thereby significantly reduce the risk of thromboembolism. To attenuate and delay the progression of symptoms in people with heart failure (HF), a device-based therapy, interatrial shunt device (IASD) implantation has been developed these years [9–13]. The rationale for the IASD is to percutaneously create an iatrogenic shunt, which then reduces the pressure of the atrial and alleviates heart failure. However, the previous implantation of an IASD will also bring challenges for future LAAC. Here we reported a case of LAAC in a patient carrying a previously implanted IASD for the first time.

## Case present

**Fig. 1:**
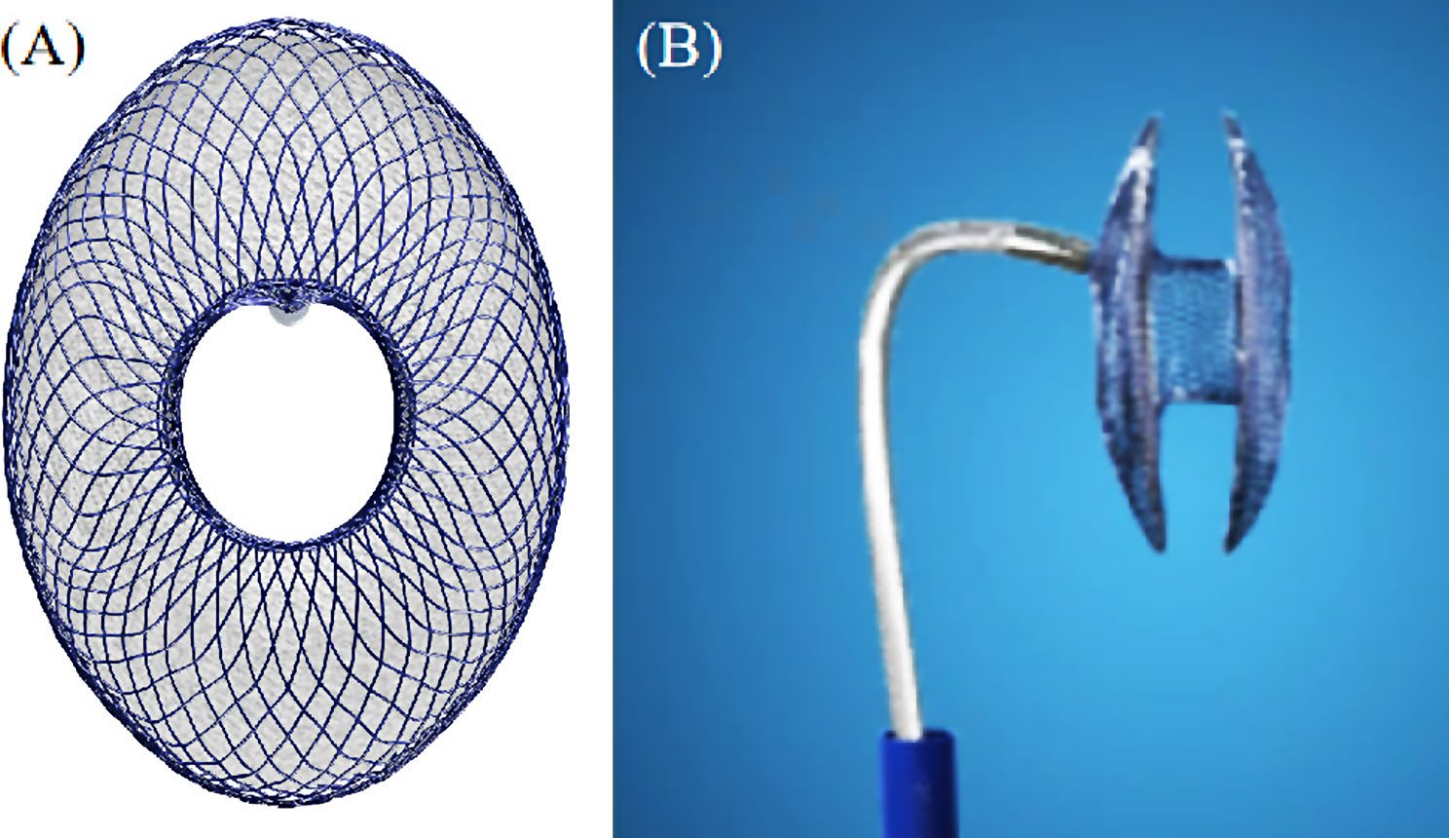
The FreeFlow® device has a “double elliptical discs, one hole” structure weaved by Nickel-titanium alloy wires, and a 5 mm diameter iatrogenic shunt. (**A**) Front view. (**B**) Side view

A 71-year‐old man diagnosed with paroxysmal AF and HF with a New York Heart Association (NYHA) class of II was admitted to our hospital. He presented a history of hypertension for about 10 years and coronary heart disease for about 5 years (coronary computerized tomography [CT] angiography can be found in supplement Fig. 1). He also reported experiencing renal dysfunction and having undergone stent implantation for aortic dissections (CT examination can be found in supplement Fig. 2). As his CHA_2_DS_2_-VASc score was 3 (hypertension, vascular disease, and age between 65 and 74), he had a strong indication for anticoagulation. However, the patient has experienced nose bleeding and gastrointestinal bleeding using warfarin or rivaroxaban with a HAS-BLED score of 4 (hypertension, abnormal function of the kidney, bleeding history or tendency, and age > 65 years). Therefore, LAAC was considered for him because he could not tolerate long-term anticoagulation. Three months ago, the patient was admitted to our hospital complaining about chest tightness and palpitations for two years. He was diagnosed with HF with a NYHA class of III and left ventricular ejection faction of 55%, pulmonary hypertension with a pulmonary artery systolic pressure of 53 mmHg, and AF. A successful IASD FreeFlow® (Con Flow MedTec, Shanghai, China) implantation then was performed followed by the usage of spironolactone (20 mg, qd), torasemide (10 mg, qd), and sacubitril/valsartan (50 mg, bid). However, the previous IASD implantation led to more difficulties for LAAC. After obtaining informed consent for the procedure, LAAC was planned to be performed using transesophageal echocardiography (TEE) guidance under local anesthesia. The FreeFlow® percutaneous atrial septal shunt system is a novel ISAD device which has a “double elliptical discs and one hole” structure weaved by Nickel-Titanium alloy wires (Fig. 1). We have performed 20 successful implantations of this device at our hospital. In this case, a 5 mm diameter iatrogenic shunt of the atrial interatrial septum was made after the implantation of FreeFlow®.

**Fig. 2:**
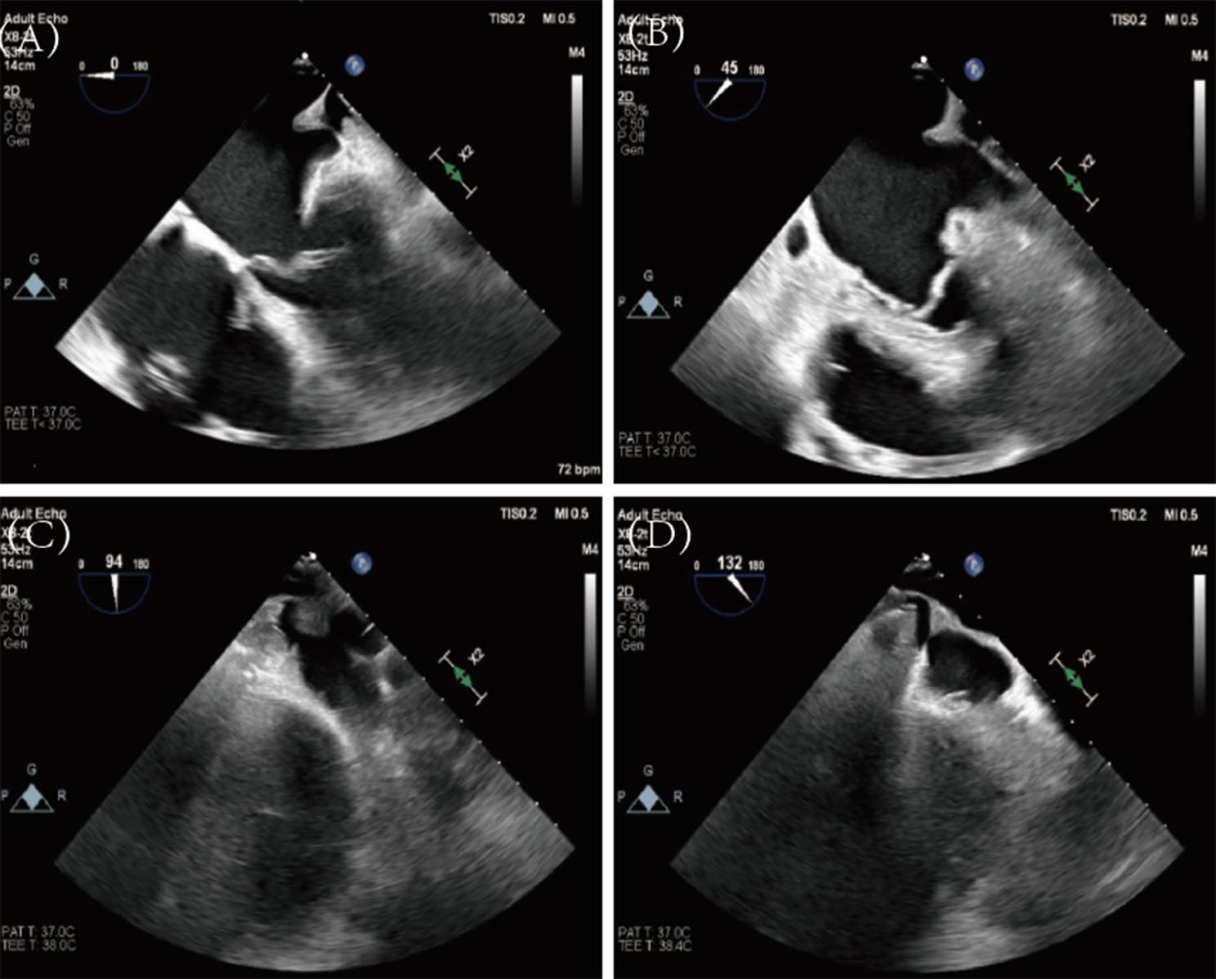
TEE examination showed a “Cauliflower” kind LAA morphology (**A**, **B**, **C**, and **D**)

During the TTE examination, a “Cauliflower” kind left atrial appendage (LAA) morphology with a large interior space was observed. The opening of LAA was oval with a slightly longer upper margin, and a shorter lower margin which almost coincided with the left atrial wall. The internal comb-like muscle was well developed inside LAA. Thematic stasis was observed with a slow emptying rate of 15 cm/s in LAA. Width and depth of LAA were measured under TEE (Fig. 2). Besides, the IASD was regularly in site, and an atrial shunt was observed.

**Fig. 3:**
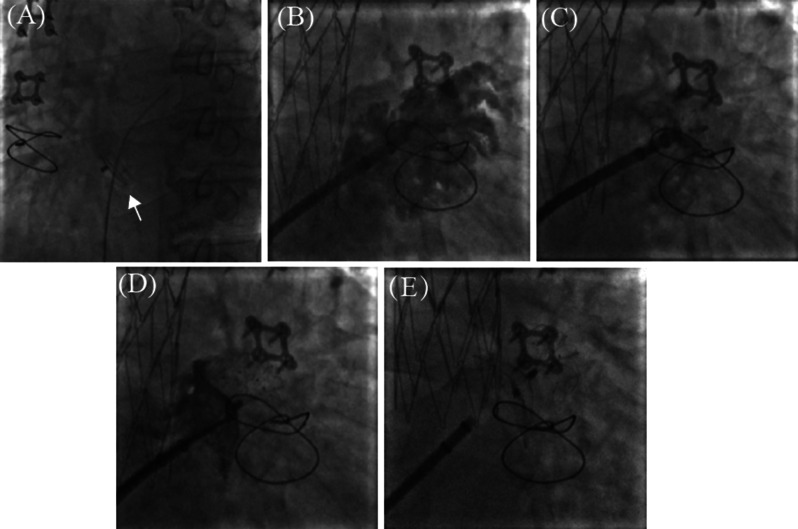
Fluoroscopy images showed the procedure of LAAC. (**A**) MPA catheter passed atrial septal through the ISAD. (**B**) Contrast was injected into LAA. (**C**) A 30–36 mm LAmbre occluder anchored appropriately. (**D**) The external disc of the occluder was opened. (**E**) Occluder was released after confirmation of proper positioning

The procedure was performed under X-ray fluoroscopy and local anesthesia. Right femoral venous access was used and we delivered a multipurpose angiography (MPA) catheter to pass the atrial septum through the iatrogenic shunt (center hole) of IASD (Fig. 3A). And then we advanced the delivery sheath into the left atrium through the IASD. After that, we used a pigtail catheter to engage the LAA through the delivery sheath. Under the fluoroscopy projection of RAO 30°, CAU 20°, the operation of LAAC was performed. A 30–36 mm LAmbre occluder was then selected according to the measurement of LAA and it was delivered to LAA, contrast was injected into LAA (Fig. 3B). The anchor plate was opened in a proper position (Fig. 3C). After we found it anchoring appropriately, the external disc of the occluder was then opened (Fig. 3D). Then the upper edge of the occluder was pulled out from LAA and we could find that the LAmbre device was deployed correctly under angiograms (Fig. 3E). Position and compression of the LAmbre device were accessed. No peri-device leak was observed under post-operative TEE examination.

## Discussion

Herein, for the first time, we report the technical feasibility of LAAC operation in patients who previously underwent IASD implantation, which hasn’t been reported before. For some patients carrying a previously implanted IASD, percutaneous LAAC may be warranted in the future. Patients who undergo the IASD implantation to attenuate HF are usually elderly patients, and they suffer from other cardiovascular complications, including arrhythmia, hypertension, and coronary heart disease, contributing to high CHA_2_DS_2_-VASc and HAS-BLED score. Therefore, with the increasing cases of IASD implantation, LAAC therapy should be considered for some of these patients.

The structural changes of atrial septal induced by IASD implantation can result in various challenges during future LAAC. Firstly, it was difficult to choose the proper puncture position because the area of the atrial septum was partially occupied by the IASD device. However, according to the IASD (FreeFlow®) implantation, a 5 mm iatrogenic shunt of atrial interatrial septum was made, it provided a channel for left atrial access operations. FreeFlow® is characterized by rapid endothelialization, so it fixed firmly to the atrial septal three months after implantation. Therefore, we decided to perform LAAC through ISAD access. Owing to this approach, unnecessary punctures were avoided resulting in less damage. Besides, for LAA occlusion, the transseptal puncture should be low, in the mid to anterior septum to facilitate engagement of the LAA. Most of the IASDs are implanted in the mid of atrial septum, the subsequent poor coaxiality of delivery sheath would affect the process of closure. In this case, the position of IASD was not high, and the coaxiality was not bad. Additionally, the LAmbre device would also obtain satisfactory results for cases with poor coaxiality [14–16]. Accordingly, in cases of percutaneous LAAC in patients with previous IASD implantation, an adjustable delivery sheath will facilitate the operation and become a better option. Moreover, patient with IASD implantation always have expansion left atrium and LAA. These enlarged chambers have deeper internal spaces, making it hard for axial localization. Consequently, overly deep placement of the device can lead to incomplete occlusion and must be avoided. Inohara et al. [17]. reported first LAmbre Closure system implantation experience in North America and also suggested that it could be considered for very large LAAs. In addition, several left atrial accesses operative strategies have been performed in different kind of interatrial devices. For example, with the guidance of intracardiac echocardiography, transseptal puncture catheterization for AF ablation is reported to be a safe and feasible procedure when performed through the native septum adjacent to the amplatzer septal occluder device [18]. Therefore, intracardiac echocardiography may also reduce the difficulty of intervention through the ISAD. Taken together, due to the implantation of IASD, the subsequent change of interatrial septum would affect the operation of LAAC. However, it can be performed safely and conveniently using the IASD access with a LAmbre Closure system.

## Conclusion

To the best of our knowledge, this is the first reported case of LAAC in patient who previously underwent IASD implantation and from this case, we confirm its technical feasibility. Therefore, for those who develop AF after receiving IVSD implantation and cannot tolerate continuous oral anticoagulants, LAAC can be considered.

### Electronic supplementary material

Below is the link to the electronic supplementary material.

Supplementary Material 1

Supplementary Material 2

Supplementary Material 3

Supplementary Material 4

Supplementary Material 5

Supplementary Material 6

Supplementary Material 7

## Data Availability

Data sharing not applicable to this article as no datasets were generated or analyzed during the current study. ETHICS DECLARATIONS.
