# Supplementary figures and images for: Left atrial appendage closure in a patient previously implanted with an interatrial shunt device: a case report
Source: BMC Cardiovasc Disord. 2024 Jun 6;24:293. doi: 10.1186/s12872-024-03904-0 (PMC11155087; doi:10.1186/s12872-024-03904-0)

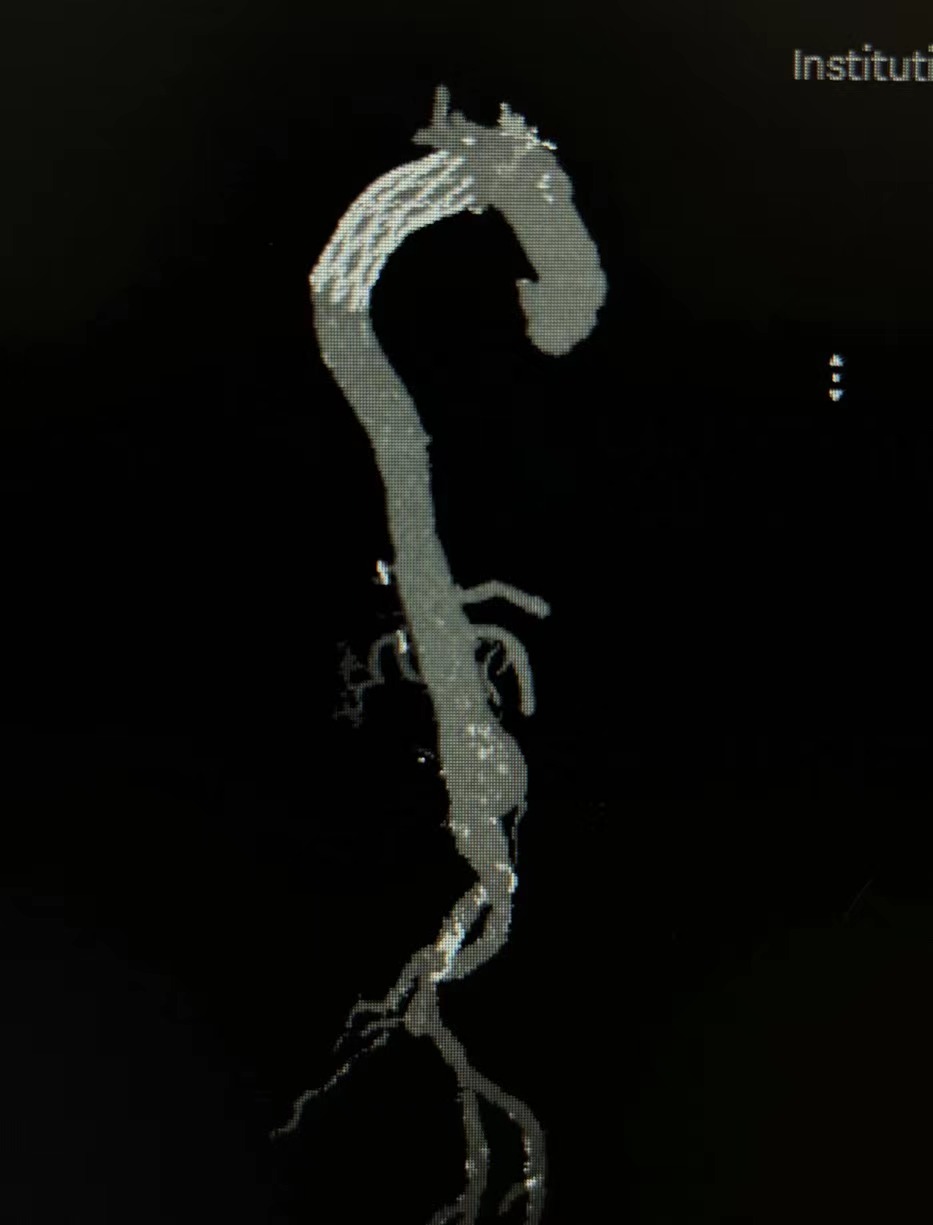

Supplement: Supplementary file 6 — Supplementary Material 6 [file 12872_2024_3904_MOESM6_ESM.png]

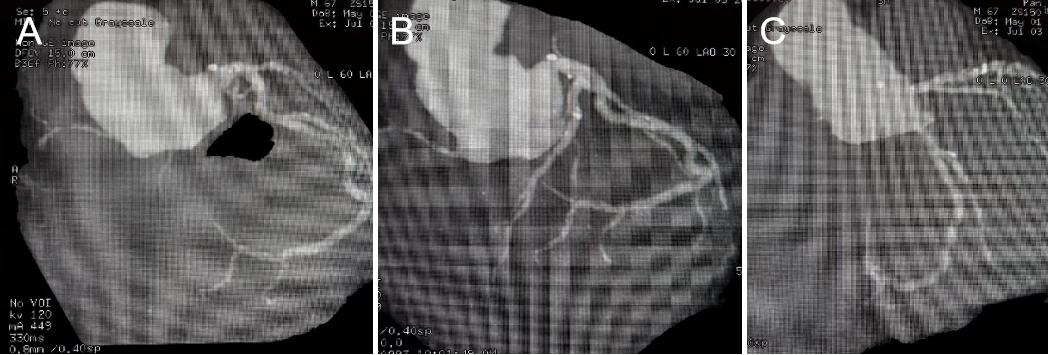

Supplement: Supplementary file 7 — Supplementary Material 7 [file 12872_2024_3904_MOESM7_ESM.png]
